# Immune priming using DC- and T cell-targeting gene therapy sensitizes both treated and distant B16 tumors to checkpoint inhibition

**DOI:** 10.1016/j.omto.2022.01.003

**Published:** 2022-01-10

**Authors:** Jessica Wenthe, Sedigheh Naseri, Ann-Charlotte Hellström, Rafael Moreno, Gustav Ullenhag, Ramon Alemany, Tanja Lövgren, Emma Eriksson, Angelica Loskog

**Affiliations:** 1 Uppsala University, Department of Immunology, Genetics and Pathology, Science for Life Laboratory, 751 85 Uppsala, Sweden; 2 IDIBELL-Institute Català d'Oncologia, 08908 L'Hospitalet de Llobregat, Barcelona, Spain; 3 Uppsala University Hospital, Department of Oncology, 751 85 Uppsala, Sweden; 4 Lokon Pharma AB, 753 20 Uppsala, Sweden

**Keywords:** oncolytic adenovirus, CD40L, 4-1BBL, immune checkpoint blockade, checkpoint inhibitor, PD-1, PD-L1, TIM-3

## Abstract

Immune checkpoint inhibitors have revolutionized the treatment of metastatic melanoma, but most tumors show resistance. Resistance is connected to a non-T cell inflamed phenotype partially caused by a lack of functional dendritic cells (DCs) that are crucial for T cell priming. Herein, we investigated whether the adenoviral gene vehicle mLOAd703 carrying both DC- and T cell-activating genes can lead to inflammation in a B16-CD46 model and thereby overcome resistance to checkpoint inhibition therapy. B16-CD46 cells were injected subcutaneously in one or both flanks of immunocompetent C57BL/6J mice. mLOAd703 treatments were given intratumorally alone or in combination with intraperitoneal checkpoint inhibition therapy (anti-PD-1, anti-PD-L1, or anti-TIM-3). Tumor, lymph node, spleen, and serum samples were analyzed for the presence of immune cells and cytokines/chemokines. B16-CD46 tumors were non-inflamed and resistant to checkpoint blockade. In contrast, mLOAd703 treatment led to infiltration of the tumor by CD8^+^ T cells, natural killer (NK) cells, and CD103^+^ DCs, accompanied by a systemic increase of pro-inflammatory cytokines interferon γ (IFN-γ), tumor necrosis factor alpha (TNF-α), and interleukin-27 (IL-27). This response was even more pronounced after combining the virus with checkpoint therapy, in particular with anti-PD-L1 and anti-TIM-3, leading to further reduced tumor growth in injected lesions. Moreover, anti-PD-L1 combination also facilitated abscopal responses in non-injected lesions.

## Graphical abstract

**Figure:**
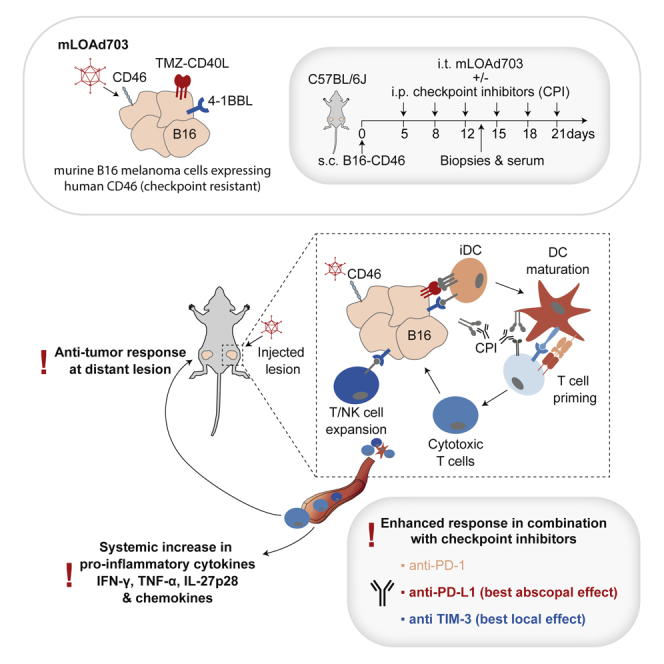

## Teaser

Many patients do not respond to checkpoint inhibitor therapy. To facilitate a response, one can utilize oncolytic viruses that induce inflammation in the tumor. The oncolytic virus, LOAd703, expresses immunostimulatory transgenes that activate crucial immune cells and can likely sensitize patients for checkpoint inhibitor therapy targeting different checkpoint molecules.

## Introduction

Malignant melanoma is an aggressive skin cancer with rising incident rates worldwide. Novel treatment options, including immune checkpoint inhibitors targeting PD-1/PD-L1, have dramatically changed the prospects for advanced stage melanoma patients, leading to both prolonged survival and complete responses.^1^ Single checkpoint inhibition therapy targeting PD-1 in melanoma patients leads to response rates of 33%–44%.^2^, ^3^, ^4^ Hence, there is a primary resistance to checkpoint inhibition therapy in most patients. Further, in patients with tumors that initially respond to treatment, the tumors often become resistant over time (acquired resistance).^5^ Primary resistance is connected to a lack of pre-existing T cells in the tumor microenvironment (TME). This can be due to reduced T cell trafficking to the tumor and/or impaired dendritic cell (DC) function, leading to inadequate co-stimulatory signaling during presentation of tumor-associated antigens to T cells. In addition, tumors may be poorly immunogenic and not generate any tumor-antigen specific T cell responses. Acquired resistance is mediated by mutations leading to interferon γ (IFN-γ) insensitivity and the loss of β-2-microglobulin, which results in the loss of major histocompatibility complex class I (MHC class I) expression and restriction of CD8^+^ T cell responses.^6^ Immune priming of therapy-resistant patients is likely required to combat these resistance mechanisms and increase responses to checkpoint inhibitors.

A promising immunotherapy approach is the use of oncolytic viruses, which can lead to direct tumor lysis with release of tumor antigens and to the recruitment and activation of pro-inflammatory immune cells. In addition, oncolytic viruses are commonly engineered into gene vehicles that carry immunostimulatory transgenes to the TME to enhance the induction of an anti-tumor immune response.^7^ Thus far, most such viruses encoded single genes, such as granulocyte-macrophage colony-stimulating factor (GM-CSF) and IFN.^8^ LOAd703 is an oncolytic serotype 5/35 adenovirus that encodes two immunostimulatory genes aiming to simultaneously activate DCs and T cells (trimerized membrane-bound [TMZ]-CD40L and 4-1BBL). In previous studies, we have demonstrated *in vitro* that LOAd703, but not the oncolytic control virus without transgenes, modulated the TME of pancreatic cancer models and induced DC as well as both natural killer (NK) cell and T cell activation.^9^ Furthermore, we have recently shown that LOAd703 can promote chimeric antigen receptor (CAR) T cell responses in B cell lymphoma models.^10^ LOAd703 is currently in clinical investigation in various cancer types as the first oncolytic virus with two immunostimulatory payloads that can express the transgenes in both tumor and stroma (ClinicalTrials.gov Identifiers: NCT02705196, NCT03225989, NCT02705196, NCT03555149). Hence, LOAd703 is a promising candidate for priming patients before checkpoint inhibitor therapy not only because of its targeting of both DC and T cell activation, but also because it could potentially overcome multiple resistance mechanisms in the TME by hijacking the transcription machinery and reducing tumor-promoting gene expression.^9^

Herein, we investigated the capacity of mLOAd703, expressing murine transgenes, to facilitate checkpoint inhibition therapy (anti-PD-1, anti-PD-L1, and anti-TIM-3) in the murine B16 melanoma model, in which resistance to checkpoint inhibitor monotherapy has frequently been demonstrated.^11^, ^12^, ^13^, ^14^, ^15^, ^16^ As mLOAd703 infection is mediated by human CD46, B16 cells previously modified by Fleischli et al. to express CD46^17^ were used in this study. Indeed, the B16-CD46 model was resistant to checkpoint inhibitor monotherapy, whereas mLOAd703 alone could hamper tumor growth and induce an immune response as shown by increased immune cell infiltration in the tumor and elevated cytokine and chemokine levels in the serum. This response was further enhanced in combination with any of the investigated checkpoint inhibitors. In particular, the combination with anti-PD-L1 resulted in a strong response with abscopal effects in a twin-tumor model.

## Results

### LOAd virus infection and subsequent transgene expression in B16-CD46 cells

**Figure 1:**
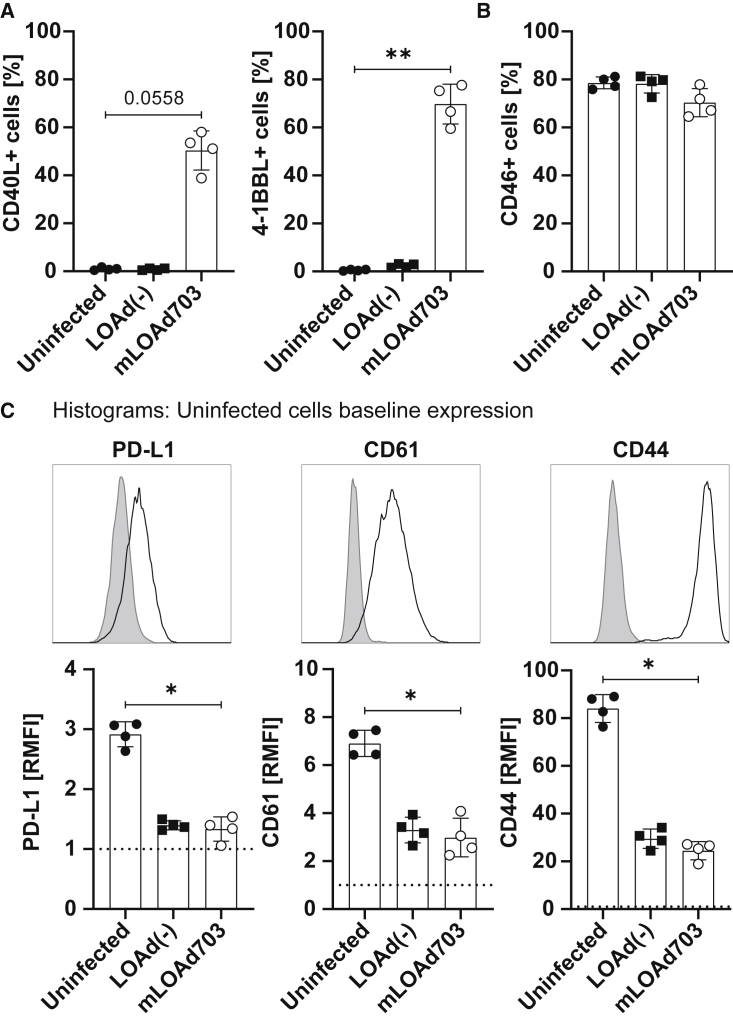
mLOAd703 infection in B16-CD46 cells *in vitro*. B16-CD46 cells were infected with LOAd(−) or mLOAd703 (50 FFU/cell) or left uninfected (A–C) Cells were analyzed for expression of the transgenes CD40L and 4-1BBL (A), CD46 (B), and tumor-promoting factors PD-L1, CD61, and CD44 (C) 48 h after infection by flow cytometry. Bar graphs in (A) and (B) show the percentage of positive cells. Histogram overlays in (C) show the baseline expression of uninfected cells (filled gray histograms: isotype control antibody; black line: staining of marker), and bar graphs display the relative mean fluorescent intensity (RMFI: fold change over isotype control). All bar graphs show mean ± SD (n = 4). Statistical differences were calculated with Kruskal-Wallis test followed by Dunn's multiple comparison test (∗p > 0.05, ∗∗p > 0.01).

The adenoviral gene vehicle mLOAd703 expressing murine TMZ-CD40L and 4-1BBL was investigated in a murine B16-CD46 melanoma model. The B16 cells are modified to express human CD46 to facilitate infection of adenoviruses with a serotype 35 fiber such as LOAd703 virus.^17^ The expression of human CD46 by the tumor is absolutely necessary for achieving a therapeutic effect by mLOAd703, which was also confirmed by the fact that no treatment effect was achieved in several other murine tumor models and a hamster tumor model, which all lack CD46 expression (Figures S1 and S2). Also, infection of parental B16-F1 cells *in vitro* leads only to somewhat comparable transgene expression when a very high viral load is used that is impossible to achieve *in vivo* (Figure S3). Hence, the effect of transgenes cannot be evaluated in the parental cell line lacking CD46 expression. We confirmed expression of human CD46 in the B16-CD46 cells over time during *in vitro* culture (Figure S4A). To evaluate if B16-CD46 cells were indeed susceptible to virus infection, cells were infected with LOAd(−) and mLOAd703 and analyzed for transgene expression by flow cytometry. Infection with mLOAd703 induced expression of both CD40L (∼50%positive) and 4-1BBL (∼70% positive) (Figure 1A), but did not significantly alter CD46 levels (Figure 1B). B16-CD46 cells express the immune checkpoint ligand PD-L1, but infection with LOAd virus downregulated the expression level *in vitro*. As we have previously observed that LOAd infection can result in reduced expression levels of genes promoting tumor growth,^9^ we explored whether two adhesion molecules that are implicated in B16 metastasis, CD61 (integrin β3) and CD44,^18^ are affected by LOAd infection. Both molecules were highly expressed on B16-CD46 cells, but their expression was decreased in cells infected with the control virus without transgenes (LOAd(−) and mLOAd703) (Figure 1C). In contrast to the control virus, the immunostimulatory capacity of the transgenes to stimulate DCs, NK cells, and T cells has previously been shown in human *in vitro* models.^9^ Likewise, murine splenocytes that were co-cultured with murine tumor cells expressing the murine transgenes after LOAd infection, but not with tumor cells infected with the control virus, have been shown to be stimulated and express a variety of cytokines (see Eriksson et al.^19^ and Figure S5). Hence, only mLOAd703 expressing the transgenes was used for *in vivo* experiments to investigate the immunostimulatory effect and combination with immune checkpoint inhibitors.

### Combination of mLOAd703 and immune checkpoint inhibitors delays tumor growth *in vivo*

**Figure 2:**
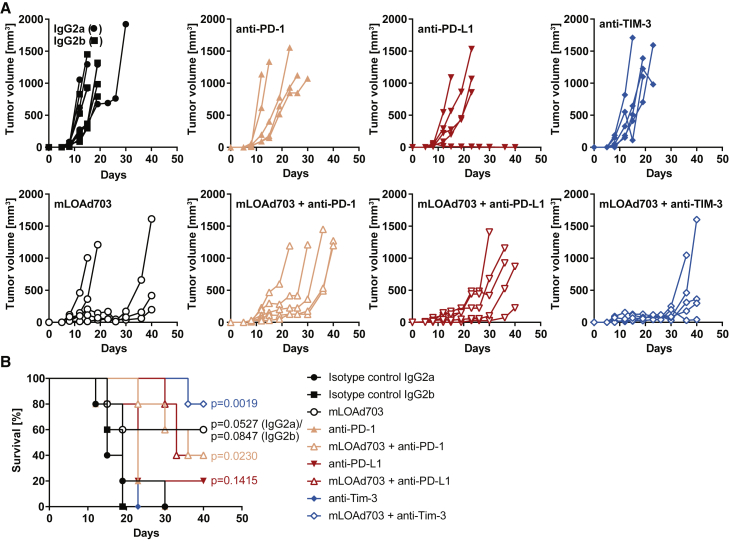
Effect of mLOAd703/immune checkpoint inhibitor treatment on B16-CD46 tumor growth *in vivo* (A and B) B16-CD46 cells (2 × 10^5^) were injected subcutaneously in syngeneic C57BL/6J mice (n = 5 per group). Treatments were initiated 5 days after tumor injection. Mice were either treated alone with mLOAd703 (intratumoral [i.t.] 1 × 10^9^ FFU/mouse), anti-PD-1, anti-PD-L1, anti-TIM-3, or IgG2a/IgG2b isotype control antibodies (intraperitoneal [i.p.] 100 μg/mouse) or treated with the combination of mLOAd703 with checkpoint antibodies for a total of six treatments over 3 weeks. (A) Graphs display individual tumor growth curves from each mouse per group. (B) Kaplan-Meier survival curves.

Next, we tested the growth of B16-CD46 cells *in vivo* (Figure S4B) and analyzed the expression of CD46 in tumor biopsies 17 days after tumor injection (Figure S4C). At that time point, almost no or very little expression of CD46 could be detected, indicating either that CD46 is lost over time *in vivo* or that the negative clones have a growth advantage *in vivo* due to increased genetic load or immunogenicity of the human molecule. Nevertheless, we investigated the response to mLOAd703 therapy in the B16-CD46 model, as we have no other immunocompetent model available and we anticipated that initial CD46 expression may still enable therapeutic responses. In addition, we aimed to determine if mLOAd703 therapy can facilitate immune checkpoint inhibition therapy. B16-CD46 cells were injected subcutaneously into immunocompetent syngeneic C57BL/6J mice, and treatments were started 5 days later. Mice were treated with intratumoral mLOAd703 injections (peritumoral at injection sites when tumors were not visible yet), treated with intraperitoneal administration of checkpoint inhibition antibodies (anti-PD-1, anti-PD-L1, or anti-TIM-3), or treated in combination with intratumoral mLOAd703 and systemic antibodies. As control, mice were treated with isotype control antibodies. In agreement with previous reports,^15^^,^^16^^,^^20^ monotherapy with immune checkpoint inhibitors had only a limited effect on tumor growth demonstrating primary resistance (Figure 2A). However, mLOAd703 alone could delay tumor growth in three out of five mice, and the combination with anti-PD-1, anti-PD-L1, and anti-TIM-3 delayed tumor growth in four to five out of five mice depending on the combination. Statistical analysis was performed on mean tumor growth curves at all time points compared with the respective antibody monotherapy (two-way analysis of variance [ANOVA] followed by Tukey's multiple comparison test). Only the combination of mLOAd703 with anti-TIM-3 significantly reduced tumor growth compared with the respective antibody monotherapy 2–4 weeks after treatment initiation (day 19: p = 0.023, day 23/26: p = 0.0083, day 30: p = 0.0057, day 36: p = 0.0349). In the anti-PD-L1 monotherapy group, one tumor did not grow out, and if this outlier is removed, the combination therapy also performed significantly better than anti-PD-L1 alone (day 23/26: p = 0.0281). The significance was lost at endpoint because control groups were euthanized when the tumor size reached 1,000 mm^3^, which occurs at an earlier time point than in the combination group. Nevertheless, mice treated with the combination of mLOAd703 with either anti-PD-1 or anti-TIM-3 survived significantly longer (p = 0.0023 and p = 0.0019) than mice treated with the respective antibody monotherapy (Figure 2B).

### Combination treatment increases immune cell infiltration in tumors

**Figure 3:**
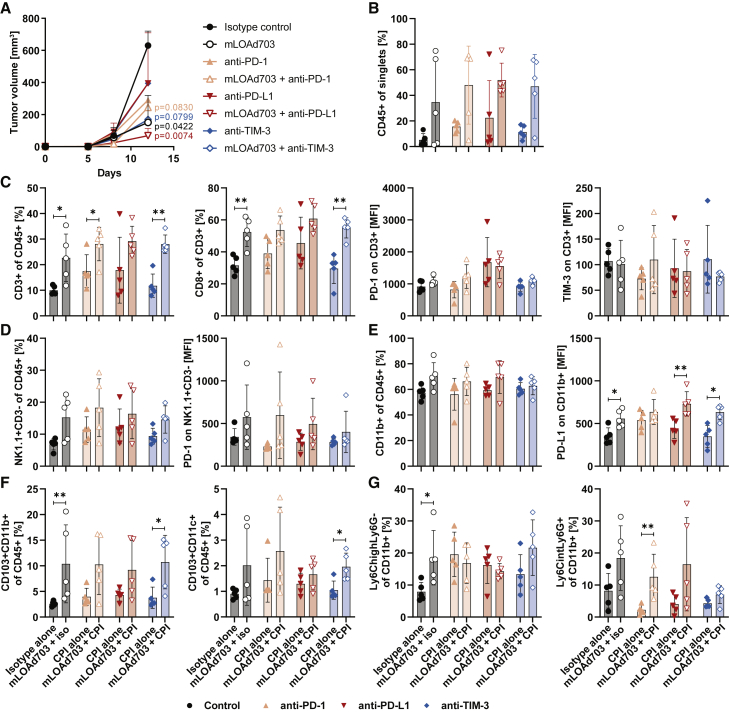
Immune cell infiltration in tumor biopsies B16-CD46 cells (2 × 10^5^) were injected subcutaneously in syngeneic C57BL/6J mice (n = 5 per group). Treatments were initiated 5 days after tumor injection. Mice were either treated alone with mLOAd703 (i.t. 1 × 10^9^ FFU/mouse), anti-PD-1, anti-PD-L1, anti-TIM-3, or IgG2a/IgG2b isotype control antibodies (i.p. 100 μg/mouse) or treated with the combination of mLOAd703 with checkpoint antibodies for a total of three treatments. One day after the third treatment (day 13), mice were sacrificed for biopsies. (A) Tumor growth curves until day 13. (B–G) Tumors were collected and single cell suspensions were prepared and stained for flow cytometry to analyze the infiltration and phenotype of immune cells: CD45^+^ immune cells (B), T cells (C), NK cells (D), myeloid cells (E), CD103^+^ DCs (F), and MDSCs (G). Bar graphs show mean ± SD (n = 5). Statistical differences between the respective single and combination treatments were calculated with two-tailed Mann-Whitney test (∗p < 0.05, ∗∗p < 0.01).

Combination of mLOAd703 with immune checkpoint inhibitors suppressed tumor growth in the otherwise checkpoint-resistant B16-CD46 tumor model. To further investigate this response, *in vivo* experiments were repeated as described above, but the mice were sacrificed 1 day after the third treatment (day 13) to collect tumor, tumor-draining lymph node (tdLN), spleen, and serum samples for analysis of the immune status. Figure 3A displays the tumor growth until endpoint showing a significant reduction in the tumor volume in mice treated with mLOAd703 compared with isotype control (p = 0.0422) and in mice treated with the combination of mLOAd703 and anti-PD-L1 compared with anti-PD-L1 alone (p = 0.0074). Samples were processed to single cell suspensions and analyzed with flow cytometry for infiltration of immune cells. The flow cytometry gating strategy is shown in Figure S6. In tumors, highest infiltration of CD45^+^ immune cells (∼50% of cells) was observed in the combination treatment groups (Figure 3B), and T cells (CD3^+^), in particular CD8^+^ T cells, were enriched in all mLOAd703-treated groups compared with the respective antibody monotherapy (Figure 3C). PD-1 expression on T cells appeared increased in either group receiving anti-PD-L1, whereas TIM-3 expression was overall unchanged (Figure 3C). The percentage of NK cells (CD3-NK1.1^+^) and expression of PD-1 on NK cells was overall highest in mice receiving mLOAd703 as either monotherapy or combination therapy (Figure 3D). Monocytes (CD11b^+^) also tended to be increased in all mLOAd703 groups, and increased PD-L1 expression was induced in mLOAd703-treated mice and with the combination of mLOAd703 with anti-PD-L1 or anti-TIM-3 compared with the respective monotherapy (Figure 3E). CD103^+^CD11b^+^ and CD103^+^CD11c^+^ DCs were overall increased in all mLOAd703-treated groups (Figure 3F). Lastly, the presence of immunosuppressive myeloid-derived suppressor cells (MDSCs) was determined (Figure 3G). Monocytic MDSCs (M-MDSCs: CD11b^+^ Ly6C^high^Ly6G^−^) tended to be increased in all treatment groups compared with isotype control and were highest upon combination of mLOAd703 with anti-TIM-3. Granulocytic/polymorphonuclear MDSCs (PMN-MDSCs: CD11b^+^ Ly6C^int^Ly6G^+^) seemed selectively enriched after treatment with mLOAd703 or the combination with either anti-PD-1 or anti-PD-L1.

### Combination treatment appears to evoke T cell migration from tumor-draining lymph nodes

**Figure 4:**
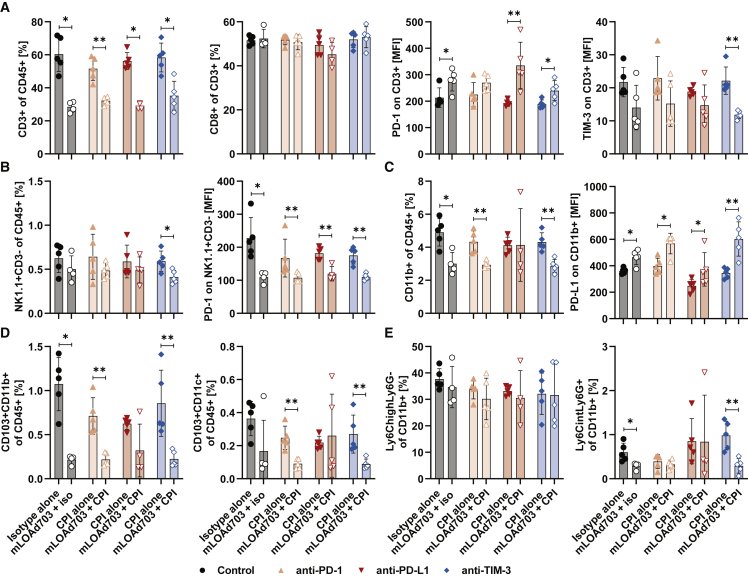
Immune cell infiltration in tumor-draining lymph nodes B16-CD46 cells (2 × 10^5^) were injected subcutaneously in syngeneic C57BL/6J mice (n = 5 per group). Treatments were initiated 5 days after tumor injection. Mice were either treated alone with mLOAd703 (i.t. 1 × 10^9^ FFU/mouse), anti-PD-1, anti-PD-L1, anti-TIM-3, or IgG2a/IgG2b isotype control antibodies (i.p. 100 μg/mouse) or treated with the combination of mLOAd703 with checkpoint antibodies for a total of three treatments. One day after the third treatment (day 13), mice were sacrificed for biopsies. (A–E) Tumor-draining lymph nodes were collected, and single cell suspensions were prepared and stained for flow cytometry to analyze the phenotype of immune cells: T cells (A), NK cells (B), myeloid cells (C), CD103^+^ DCs (D), and MDSCs (E). Bar graphs show mean ± SD (n = 4–5). Statistical differences between the respective single and combination treatments were calculated with two-tailed Mann-Whitney test (∗p < 0.05, ∗∗p < 0.01).

In the tdLNs, we noted a decrease in the percentage of T cells in the groups treated with mLOAd703, which suggests that the intratumoral mLOAd703 treatment resulted in the recruitment of T cells from the tdLN (Figure 4A). Remaining T cells in the tdLNs of mLOAd703-treated groups displayed higher expression of PD-1 and lower expression of TIM-3 compared with the respective antibody monotherapy. The percentage of NK cells in the tdLNs was overall low and slightly further reduced upon mLOAd703 treatment, accompanied by decreased expression of PD-1 on NK cells (Figure 4B). Likewise, the percentage of CD11b^+^ monocytes was significantly reduced in all mLOAd703-treated groups except for the combination with anti-PD-L1, and PD-L1 expression on monocytes was enhanced compared with the respective antibody monotherapies (Figure 4C). Both CD103^+^CD11b^+^ and CD103^+^CD11c^+^ DCs were also reduced upon mLOAd703 treatments in tdLNs (Figure 4D). No change in M-MDSCs was observed, whereas PMN-MDSCs were generally lower with anti-PD-1 treatment and reduced upon treatment with mLOAd703 and the combination with anti-TIM-3 (Figure 4E). The phenotypes of immune cells in the spleen were mostly unchanged and are shown in Figure S7. Likewise in the tdLNs, we noted a slight reduction of the percentage of T cells in all mLOAd703-treated groups. In addition, M-MDSCs tended to be reduced in particular with the combination treatments.

### Pro-inflammatory cytokines are upregulated in the serum of mice receiving combination treatment

**Figure 5:**
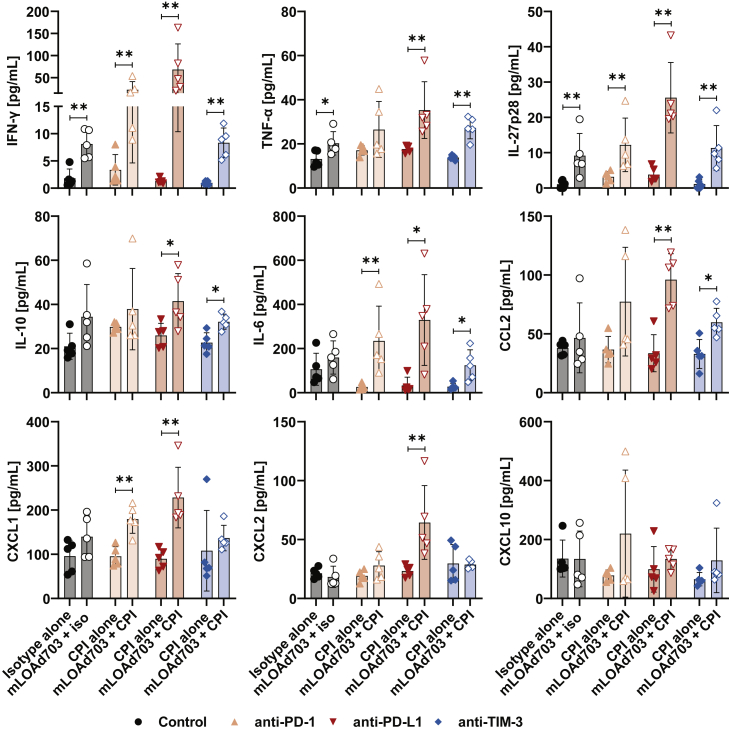
Cytokine and chemokine levels in serum B16-CD46 cells (2 × 10^5^) were injected subcutaneously in syngeneic C57BL/6J mice (n = 5 per group). Treatments were initiated 5 days after tumor injection. Mice were either treated alone with mLOAd703 (i.t. 1 × 10^9^ FFU/mouse), anti-PD-1, anti-PD-L1, anti-TIM-3, or IgG2a/IgG2b isotype control antibodies (i.p. 100 μg/mouse) or treated with the combination of mLOAd703 with checkpoint antibodies for a total of three treatments. One day after the third treatment (day 13), mice were sacrificed, and blood samples were collected. Serum was isolated and analyzed for the presence of cytokines and chemokines with V-PLEX Mouse Cytokine 29-Plex Kit. Bar graphs show mean ± SD (n = 5). Statistical differences between the respective single and combination treatments were calculated with two-tailed Mann-Whitney test (∗p < 0.05, ∗∗p < 0.01).

Analysis of tumors and tdLNs revealed an induction of an immune response by infiltration of effector immune cells into the tumor. To further investigate systemic treatment effects, serum samples were analyzed with an exploratory multiplex kit for the presence of various cytokines and chemokines (Figure 5). Treatment with mLOAd703 induced increased levels of IFN-γ, tumor necrosis factor alpha (TNF-α), and IL-27p28, but highest levels were observed with the combination of checkpoint inhibitors. In particular, the combination treatment of mLOAd703 with anti-PD-L1 significantly upregulated a multitude of different cytokines and chemokines, including IL-10, IL-6, CCL2, CXCL1, and CXCL2. CXCL10 levels were similar between the groups, but with slightly higher levels in mice receiving the combination of mLOAd703 with anti-PD-1.

### mLOAd703/anti-PD-L1 combination treatment controls tumor growth in a twin-tumor model

**Figure 6:**
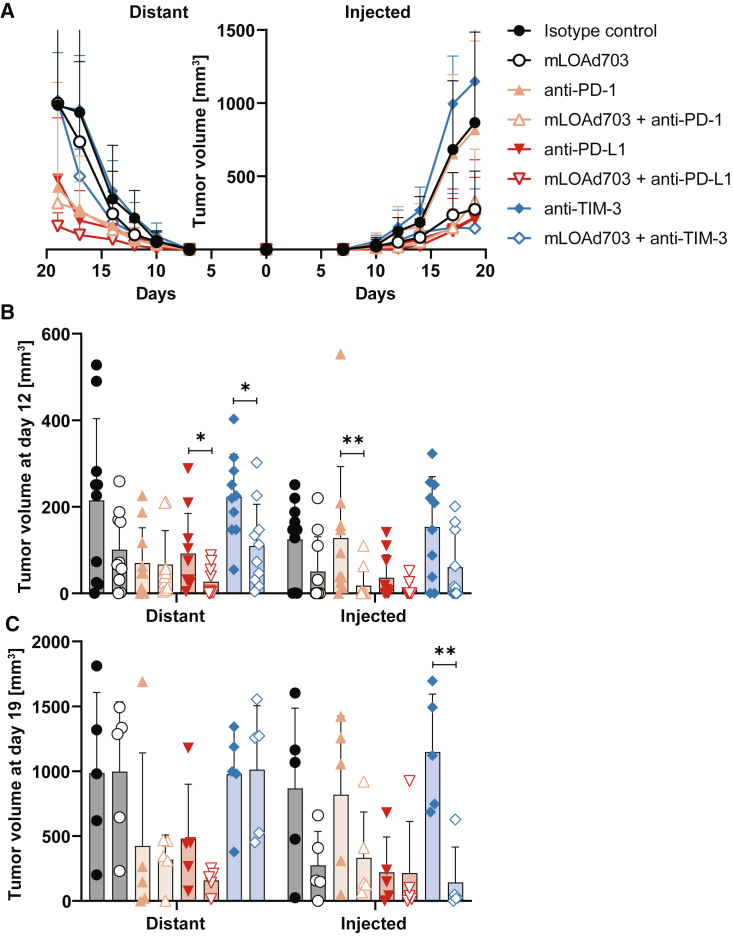
Combination of mLOAd703 and immune checkpoint inhibitors in a B16-CD46 twin-tumor model B16-CD46 cells (1 × 10^5^) were injected subcutaneously in both flanks of syngeneic C57BL/6J mice (n = 10 per group until day 14, when 5 mice per group were sacrificed for biopsies). Treatments were initiated 4 days after tumor injection. Mice were either treated with IgG2a/IgG2b isotype control antibodies, anti-PD-1, anti-PD-L1, anti-TIM-3 (i.p. 100 μg/mouse), or mLOAd703 (i.t. 1 × 10^9^ FFU/mouse) or with the combination of mLOAd703 with checkpoint inhibition antibodies for a total of six treatments over 3 weeks. (A) Mean tumor growth curves ±SD per group and tumor site (distant versus injected lesion). (B) Bar graphs in show the mean tumor volume ± SD at day 12 and 19, respectively. Statistical differences between the respective single and combination treatments were calculated with two-tailed Mann-Whitney test (∗p < 0.05, ∗∗p < 0.01).

Next, we utilized a twin-tumor model to explore whether the combination therapy of mLOAd703 with immune checkpoint inhibitors can induce systemic anti-tumor immune responses. B16-CD46 cells were injected subcutaneously at the same time into both flanks of C57BL/6J mice, and mLOAd703 treatments were given intratumorally, but only in one of the tumor lesions (right/injected lesion). Antibodies were given intraperitoneally as in the single-tumor model. Half of the mice were followed for tumor growth, and half were sacrificed at day 14 for analysis of tumor and serum samples. The tumor growth in both lesions is displayed in Figure 6A (left/distant lesion versus right/injected lesion). As observed in the single-tumor model, mLOAd703 treatment alone and in combination with checkpoint inhibitors could delay tumor growth in the injected lesion, but in contrast to the previous experiments, monotherapy with anti-PD-L1 induced an initial response in both lesions. Nevertheless, the combination of mLOAd703 and anti-PD-L1 was best in controlling the tumor growth of the distant lesion, whereas the combination with anti-TIM-3 had less effect on the distant tumor, but was most efficient in hampering the growth of the injected tumor lesion. Figures 6B and 6C show the mean tumor growth at day 12 and 19, respectively. At day 12, the tumor size of all mLOAd703-injected lesions was low, but also at this early time point the combination with anti-PD-L1 significantly reduced tumor growth of the distant lesion compared with anti-PD-L1 monotherapy. The trends remained similar at day 19, and most significant tumor control of the injected lesion was observed with the combination of mLOAd703 with anti-TIM-3. However, this combination lost its effect on the distant lesion over time.

### mLOAd703 treatment enriched CD8^+^ T cells in both injected and distant tumor lesions

**Figure 7:**
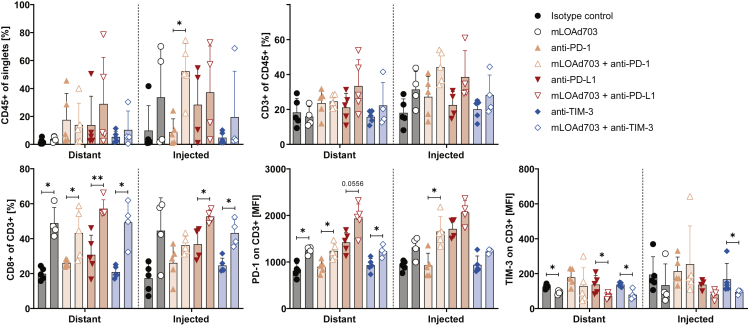
Immune cell infiltration in tumor biopsies from the B16-CD46 twin-tumor model B16-CD46 cells (1 × 10^5^) were injected subcutaneously in both flanks of syngeneic C57BL/6J mice. Treatments were initiated 4 days after tumor injection. Mice were either treated with IgG2a/IgG2b isotype control antibodies, anti-PD-1, anti-PD-L1, anti-TIM-3 (i.p. 100 μg/mouse), or mLOAd703 (i.t. 1 × 10^9^ FFU/mouse) or with the combination of mLOAd703 with checkpoint inhibition antibodies for a total of four treatments before 5 mice per group were sacrificed (day 14) for biopsies. Tumors from both injected and distant lesions were collected, and single cell suspensions were prepared and stained for flow cytometry to analyze the infiltration and phenotype of T cells. Bar graphs show mean ± SD (n = 4–5). Statistical differences between the respective single and combination treatments were calculated with two-tailed Mann-Whitney test (∗p < 0.05, ∗∗p < 0.01).

Half of the mice were sacrificed 1 day after four treatments (day 14), and both tumor lesions were collected and analyzed for T cell infiltration with flow cytometry (Figure 7). At this time point, no tumor was detectable in six mice and thus could not be analyzed (no tumors in injected/right lesions: 1× anti-PD-L1, 1× mLOAd703, 1× mLOAd703 + anti-PD-L1, 1× mLOAd703 + anti-TIM-3, no tumors in distant/left lesions: 1× mLOAd703, 1× mLOAd703 + anti-TIM-3). Infiltration of CD45^+^ immune cells was overall lower and more spread in the twin-tumor model compared with the single-tumor model. Highest immune cell infiltration in the injected lesion was noted with mLOAd703/anti-PD-1 combination, and two out of the four injected lesions treated with mLOAd703 alone or together with anti-PD-L1 also showed similar high immune cell infiltration. Interestingly, this infiltration was also increased in two of the distant lesions of the anti-PD-L1 combination group. The percentage of T cells within the immune cell compartment tended to be increased in the injected lesions of all groups receiving mLOAd703. The combination of mLOAd703 with anti-PD-L1 also appeared to increase T cells in the distant lesion. This abscopal effect on the distant lesion was more pronounced for CD8^+^ T cells, which were significantly enriched in all distant lesions upon mLOAd703 treatment, with highest levels upon anti-PD-L1 combination. Likewise, PD-1 expression was significantly increased on T cells in distant lesions upon mLOAd703 treatment, especially when combined with anti-PD-L1, indicating a systemic T cell activation. In contrast, TIM-3 was only minimally expressed on T cells in both lesions and overall reduced upon mLOAd703 treatment.

### Levels of IFN-γ, TNF-α, and IL-27p28 in serum are highest in mice receiving combination treatment in twin-tumor model

**Figure 8:**
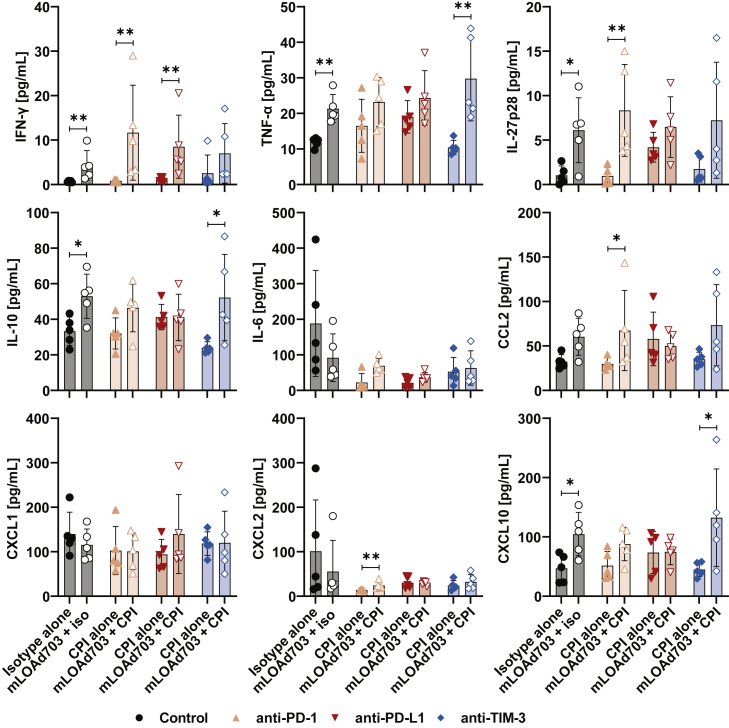
Cytokine and chemokine levels in serum samples from the B16-CD46 twin-tumor model B16-CD46 cells (1 × 10^5^) were injected subcutaneously in both flanks of syngeneic C57BL/6J mice. Treatments were initiated 4 days after tumor injection. Mice were either treated with IgG2a/IgG2b isotype control antibodies, anti-PD-1, anti-PD-L1, anti-TIM-3 (i.p. 100 μg/mouse), or mLOAd703 (i.t. 1 × 10^9^ FFU/mouse) or with the combination of mLOAd703 with checkpoint inhibition antibodies for a total of four treatments before 5 mice per group were sacrificed (day 14), and blood samples were collected. Serum was isolated from blood samples and analyzed for the presence of cytokines and chemokines with V-PLEX Mouse Cytokine 29-Plex Kit. Bar graphs show mean ± SD (n = 5). Statistical differences between the respective single and combination treatments were calculated with two-tailed Mann-Whitney test (∗p < 0.05, ∗∗p < 0.01).

To investigate further systemic effects, serum samples were analyzed with the same multiplex assay as in the single-tumor model (Figure 8). Overall, cytokine levels were lower in the twin-tumor model, but all treatments with mLOAd703 resulted in higher levels of IFN-γ, TNF-α, and IL-27p28 as observed before. In contrast to the single-tumor model, the highest IL-6 level was detected in mice treated with isotype control antibodies and was reduced in all active treatment groups. The chemokine CXCL2 was reduced in a similar manner, whereas levels of CXCL1 were overall unchanged. CCL2 and CXCL10 were mostly increased in any group receiving mLOAd703 treatment, but the highest increase in CXCL10 was observed in combination with anti-TIM-3.

## Discussion

Metastatic melanoma treatment has been revolutionized by immune checkpoint inhibition therapy, but a substantial number of tumors show no response, or treatment resistance is established over time.^6^^,^^21^ PD-1/PD-L1 blockade aims to restore the function of anergic tumor-infiltrating T cells, thereby inducing an efficient anti-tumor response. Thus, the presence of T cells in the tumors is essential to benefit from checkpoint inhibition.^22^ Oncolytic viruses are currently tested in multiple clinical trials as a combination therapy with checkpoint inhibitors.^23^ For instance, the combination of talimogene laherparepvec, an oncolytic herpes virus expressing GM-CSF, with pembrolizumab increased the objective response rates to 62% in patients with metastatic melanoma.^24^ Herein, we explored the use of the oncolytic adenovirus LOAd703 in combination with anti-PD-1, anti-PD-L1, or anti-TIM-3 checkpoint blockade in a syngeneic immunocompetent B16-CD46 murine model. In addition to its oncolytic function, LOAd703 has been genetically engineered to express the strong T helper 1 (Th1) response-promoting transgenes TMZ-CD40L and 4-1BBL, both in tumor cells and in the stroma, to promote activation of DCs and T cells.^9^ Hence, LOAd703 may induce inflammation in the tumor lesion more effectively than other similar viruses because the stroma can participate to express TMZ-CD40L and 4-1BBL, while tumor cells may be killed by oncolysis, which would eventually reduce transgene expression time.

There are several hurdles for *in vivo* modeling of LOAd703. First, murine cells are non-permissive for adenovirus replication,^25^ and thus no oncolysis-related treatment effects can be evaluated. Second, a murine version of LOAd703 expressing the respective murine transgenes (mLOAd703) must be used, as the human transgenes have no or only limited cross-reaction to their murine counterparts. Third, LOAd viruses are chimeric serotype 5/35 adenoviruses and thus infect cells via human CD46, which has no sufficient homolog in the mouse system, and as a result LOAd viruses have no treatment effect on murine tumors. To overcome this last hurdle, we utilized a murine B16 cell line modified to express human CD46,^17^ which enables mLOAd703 infection and subsequent transgene expression in the tumor cells but not in the surrounding tumor stroma. B16-CD46 cells stably expressed CD46 *in vitro* and enabled efficient mLOAd703 infection as seen by the induced expression of CD40L and 4-1BBL. B16-CD46 cells are PD-L1^+^ and express high levels of the adhesion molecules CD61 and CD44, which are both implicated in the metastatic process of B16 melanoma.^26^^,^^27^ Interestingly, these tumor-promoting factors were reduced upon virus infection *in vitro*, which might be due to the viral interaction with the cell's transcription machinery. Subcutaneously injected B16-CD46 cells formed tumors; however, CD46 expression was severely reduced in resected tumors at day 17, which may be due to increased immunogenicity of the CD46^+^ clones, as human CD46 may serve as a tumor antigen in this model. The loss of CD46 likely reduces the infection efficiency and transgene expression by mLOAd703 *in vivo*, especially at later treatment time points. Despite these deficiencies of our model system, mLOAd703 monotherapy could delay tumor growth, and this delay was further enhanced in combination with checkpoint inhibitors. In contrast, monotherapy with checkpoint inhibitors had only a limited effect. This is in line with other studies, in which the resistance to checkpoint inhibitors in the B16 model could be overcome with different virotherapies.^11^, ^12^, ^13^, ^14^, ^15^ In our model, the best therapeutic effect was observed at about 2–3 weeks after treatment initiation. However, the majority of tumors eventually started to grow again, which was probably related to the loss of CD46 impairing the efficacy of repeated mLOAd73 injections and thereby prohibiting the establishment of long-term immunity.

Analysis of the immune cell infiltration in biopsies and cytokines/chemokines in serum revealed a strong stimulation of the immune system upon combination treatment, but not with checkpoint inhibitor monotherapy. In particular, CD8^+^ T cell infiltration was enhanced together with an increase in serum levels of effector cytokines IFN-γ and TNF-α. Similarly, Singh et al. have demonstrated that intratumoral injection of a replication-deficient adenovirus expressing CD40L overcame primary resistance to checkpoint blockade therapy in B16 melanoma by inducing CD8^+^ T cell responses.^14^ Furthermore, a high IFN-γ gene signature has been suggested to predict clinical outcome for PD-1/PD-L1 checkpoint therapy.^28^, ^29^, ^30^ Also, NK cells and their expression of PD-1 tended to be increased with mLOAd703 treatment. PD-1+ NK cells have been demonstrated to be highly functional, but can be impaired by PD-L1 engagement, and are involved in the therapeutic effect of PD-L1 blockade.^31^^,^^32^ NK cell stimulation is presumably mediated by 4-1BBL transgene expression in our model, as 4-1BBL is a potent activator of both T and NK cells.^33^ Facilitating NK cell responses may be crucial in patients who acquire a loss of MHC class I molecules on tumor cells, which is frequently seen in melanoma patients no longer benefiting from immune checkpoint inhibitors.^34^

DCs have emerged as major players in checkpoint blockade therapy. PD-L1 expression on immune cells, in particular on DCs and macrophages, has been acknowledged to extensively contribute to the evasion of the anti-tumor immune response^35^ and predicts clinical responses better than PD-L1 expression on tumor cells.^20^^,^^36^ We noted an upregulation of PD-L1 on the myeloid cell compartment upon mLOAd703 treatment, and we have previously shown *in vitro* that LOAd703, but not the oncolytic control virus lacking transgenes, is a potent activator of DCs.^9^ In this study, CD103^+^CD11b^+^ cells were increased in mLOAd703-treated tumors. In particular, CD103^+^ cross-presenting DCs have been found to be crucial for the combination of anti-4-1BB and anti-PD-1 therapy in murine models^37^ and for the drainage of tumor antigens to the lymph node.^38^ Fransen et al. furthermore showed that the tdLNs are the main site for T cell reinvigoration.^39^ At the time point of biopsy collection, we noted a significant reduction of T cells in the tdLNs of mLOAd703-treated mice. One explanation for this might be that primed T cells had already migrated to the tumor site, which would be in agreement with the increased T cell infiltration seen in the tumor biopsy. The remaining T cells within tdLNs of mice receiving both mLOAd703 and anti-PD-L1 displayed highest PD-1 expression. This indicates that the T cells were indeed better activated in this setting, as Xiong et al. have shown that PD-1^+^ T cells are actually highly activated, rather than exhausted, and that anti-PD-L1 therapy enhances these cells.^40^

In the spleen, we noted a significant reduction of M-MDSCs in the mLOAd703/anti-PD-L1 combination treatment group, suggesting the induction of systemic treatment effects. In addition, various serum cytokines and chemokines were upregulated upon combination treatments, including the Th1 cytokines IFN-γ, TNF-α, and IL-27p28. IL-27 is secreted by antigen-presenting cells and has been shown to activate both CD4^+^ and CD8^+^ T cells.^41^ Furthermore, chemokines CCL2, CXCL1, and CXCL2 were upregulated and potentially mediated the influx of lymphocytes. For instance, CCL2 has been suggested to promote immune cell infiltration and memory responses in B16 tumors.^42^^,^^43^

To investigate systemic anti-tumor effects, a twin-tumor model was utilized, in which only one of the two tumor lesions was injected with mLOAd703. Here, combination therapy of mLOAd703 with anti-PD-L1 controlled tumor growth in the injected lesion, but also resulted in the delayed growth of the distant lesion. This was accompanied by an enrichment of CD8^+^ T cells in both tumor lesions, which were likely highly activated as seen by increased PD-1 expression. The serum levels of the investigated analytes were overall lower in the twin-tumor model. Chemokine levels were largely unchanged, although CXCL10 expression, which is associated with T cell infiltration and subsequent responses to checkpoint inhibition,^44^, ^45^, ^46^ was upregulated upon mLOAd703 monotherapy and combination with anti-TIM-3.

Overall, combination of mLOAd703 and anti-PD-L1 resulted in the most robust anti-tumor responses considering both the mLOAd703-injected tumor lesion and the distant lesion. However, the combination with anti-TIM-3 also induced potent responses at the injected tumor lesion specifically. Hence, a combination treatment of mLOAd703 with both anti-PD-L1 and anti-TIM-3 may be of interest to achieve the best overall responses. The combination of anti-PD-L1 and anti-TIM-3 has been found to be superior by others in murine tumor models that did not respond well to each monotherapy alone.^47^ Likewise, Sun et al. found that their oncolytic virus therapy showed synergistic effects only in combination with both anti-PD-1 and anti-TIM-3 therapy, but not in combination with either checkpoint inhibitor alone.^48^ Thus, it would be of interest to explore the triple combination of mLOAd703, anti-PD-L1, and anti-TIM-3 in future experiments. Moreover, further mechanistic studies encompassing neutralization experiments are needed to pinpoint the role of the different immune cells and cytokines in the anti-tumor immune response.

In conclusion, CD40/4-1BB-stimulation in tumors using mLOAd703 gene therapy induced immune priming and sensitized tumors resistant to checkpoint inhibitor treatment directed to anti-PD-1, anti-PD-L1, and anti-TIM-3. Intratumoral mLOAd703 induced abscopal effects as monotherapy, but PD-L1 checkpoint inhibition made the abscopal effects more robust as shown by increased tumor control, immune cell infiltration, and serum cytokine levels. Clinical investigation to evaluate the capacity of LOAd703 to sensitize patients to PD-L1 therapy has recently started in a pancreatic cancer trial as well as in melanoma patients refractory to previous checkpoint inhibitor treatment (NCT02705196, NCT04123470) and in a study treating patients with refractory colorectal cancer (NCT03555149).

## Materials and methods

### Cell culture

Murine B16-CD46 melanoma cell lines^17^ were a kind gift from Dr. Hemmi, University of Zurich, and were cultured in RPMI-1640 supplemented with 8% FBS, 100 U/mL penicillin and 100 μg/mL streptomycin (1% PeSt), and 1 mg/mL Geneticin. Media and supplements were purchased from Thermo Fisher Scientific, Waltham, MA, USA.

### LOAd virus construction

LOAd viruses were provided by Lokon Pharma, AB, Uppsala, Sweden. LOAd viruses are chimeric serotype 5/35 adenoviruses, which are modified to express immunostimulatory transgenes under the control of a cytomegalovirus (CMV) promoter. The generation of these viruses have been described previously,^19^ but mLOAd703 encoding for murine transgenes TMZ-CD40L and 4-1BBL was used in this study for the first time. The virus concentration was determined as fluorescent forming units (FFU)/mL and represented viable infectious viruses.

### Phenotype analysis of mLOAd703-infected B16-CD46

B16-CD46 cells were infected with LOAd(−) or mLOAd703 or left uninfected. For this, cells were washed and pelleted in serum-free medium before the respective virus (50 FFU/cell) was added to the pellet. Cells were incubated for 2 h at 37°C before complete growth medium was added for further culture. After 48 h, cells were harvested, washed in fluorescence-activated cell sorting (FACS) staining buffer (PBS supplemented with 3 mM EDTA [Thermo Fisher, Waltham, MA, USA] and 0.5% BSA [Sigma-Aldrich, Saint Louis, MO, USA]) and stained with fluorescent-labeled antibodies targeting CD40L (MR1), 4-1BBL (TSK-1), CD46 (TRA-2-10), PD-L1 (MIH7), CD61 (2C9.G2), and CD44 (IM7). Antibodies were purchased from BioLegend (San Diego, CA, USA). Stained cells were fixed in PBS containing 1% formaldehyde and 3 mM EDTA. Samples were analyzed with BD FACS Canto 2 (BD Biosciences, San Jose, CA, USA) and FlowJo software (FlowJo, Ashland, OR, USA).

### *In vivo* experiments

All animal experiments were approved by the local animal ethical review board in Uppsala, Sweden (DNr 5.8.18–13471/2017). Murine B16-CD46 melanoma cells (1–2 × 10^5^) were injected subcutaneously in one flank or simultaneously in two flanks of syngeneic 8- to 9-week-old female C57BL/6J mice purchased from Taconic, Denmark (5–10 mice per group). Treatments were initiated 4–5 days after tumor injection. Mice were either treated alone with mLOAd703 (intratumoral injection; 1 × 10^9^ FFU/mouse in 50 μL), anti-PD-1 (clone: RMP1-14), anti-PD-L1 (clone: 10F.9G2), anti-TIM-3 (clone: RMT3-23) or IgG2a/IgG2b isotype controls or treated with the combination of mLOAd703 with each checkpoint antibody. Antibodies were purchased from Bio X Cell (West Lebanon, NH, USA) and given intraperitoneally (100 μg/mouse in 100 μL). Treatments were given twice per week for a total of 3 weeks (e.g., from day 5 to day 22). The experimental time line of the *in vivo* studies is depicted in Figure S8. Tumor growth was monitored by measuring the tumor volume (ellipsoid volume: $\frac{4}{3}\times \pi \times \frac{length}{2}\times \frac{width}{2}\times \frac{height}{2}$), and mice with tumors >1,000 mm^3^ were sacrificed.

### Biopsy analysis

Mice were sacrificed 1 day after the third or fourth treatment (day 13/14 post tumor injection) and blood samples, tumors, spleens, and tdLNs were collected. For the twin tumor model, only blood samples and both tumors were collected. Serum was isolated from blood samples by allowing the blood to clot at room temperature before centrifugation at 10,000 × *g* for 5 min. Serum samples were analyzed for cytokines and chemokines with V-PLEX Mouse Cytokine 29-Plex Kit from Meso Scale Diagnostics (Rockville, MD, USA) according to manufacturer's instructions. All organs were placed on ice in PBS. Tumors were dissociated with 2 WU/mL of Liberase TL (Roche, Basel, Switzerland), and single cell suspensions were achieved by passing samples through a 70 μm cell strainer. Spleens and tdLNs were punctured with needles, and cells within the organs were released by applying pressure with the backside of a syringe plunger. Supernatants containing single cells were transferred to tubes. Splenocytes were treated with BD Pharm Lyse lysing solution to lyse red bloods. Before staining with fluorescent-labeled antibodies, all cells were washed in FACS staining buffer and Fc receptors were blocked with TruStain FcX (BioLegend). The following antibodies (BioLegend) were used for staining: anti-CD45 (30-F11), anti-CD3 (17A2), anti-CD8 (53–6.7), anti-NK1.1 (PK136), anti-CD107a (1D4B), anti-PD-1 (29F.1A12), anti-CD11b (M1/70), anti-I-A/I-E (M5/114.15.2), anti-PD-L1 (155,404), anti-Ly6C (HK1.4), anti-Ly6G (1A8), anti-CD103 (2 × 10^7^) and respective isotype controls when applicable. Samples were analyzed with BD FACS Canto 2 and FlowJo software.

### Statistics

Statistical analysis was performed with GraphPad Prism 9 (GraphPad Software, San Diego, CA, USA). For non-parametric data containing more than two groups, Kruskal-Wallis test followed by Dunn's multiple comparison test was used. To determine statistical differences of each single treatment compared with the respective combination treatment, non-parametric two-tailed Mann-Whitney test was used. Differences in tumor volumes were calculated for the last day of analysis unless stated otherwise. Kaplan-Meier survival curves were analyzed with log rank (Mantel-Cox) test.

## Supplemental information

Document S1. Figures S1–S8

Document S2. Article plus supplemental information
