# Supplementary material for: Immune priming using DC- and T cell-targeting gene therapy sensitizes both treated and distant B16 tumors to checkpoint inhibition
Source: Mol Ther Oncolytics. 2022 Jan 10;24:429–42. doi: 10.1016/j.omto.2022.01.003 (PMC8810301; doi:10.1016/j.omto.2022.01.003)
Supplement: Document S1. Figures S1–S8 [file mmc1.pdf]

OMTO, Volume *24*

Supplemental information

Immune priming using DC- and T cell-targeting gene therapy sensitizes both treated and distant B16 tumors to checkpoint inhibition

Jessica Wenthe, Sedigheh Naseri, Ann-Charlotte Hellström, Rafael Moreno, Gustav Ullenhag, Ramon Alemany, Tanja Lövgren, Emma Eriksson, and Angelica Loskog

Supplementary Figure 1

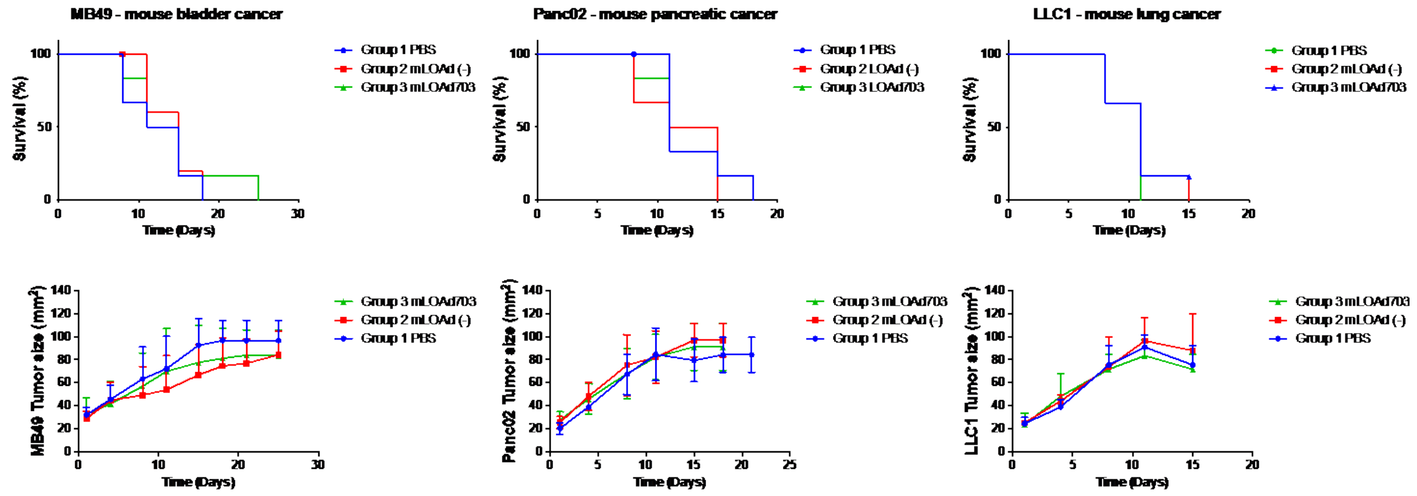

Figure S1: Treatment of syngeneic mice carrying murine tumors with no human CD46 expression. C57BL6 mice (n=6 per group) were injected with $3x10^5$ murine syngeneic tumor cells (MB49, Panc02 or LLC1). Starting 8 days after tumor cell injection, the mice were treated 6x (twice per week) with mLOAd703 ($1x10^9$ FFU) encoding murine TMZ-CD40L and 4-1BBL, LOAd(-) ($1x10^9$ FFU) without a transgene cassette or with phosphate buffered saline (PBS) as control. The treatments were given intra/peri-tumoral. The figures demonstrates the mean survival and growth rate of the tumors. Error bars represents SEM.

Supplementary Figure 2

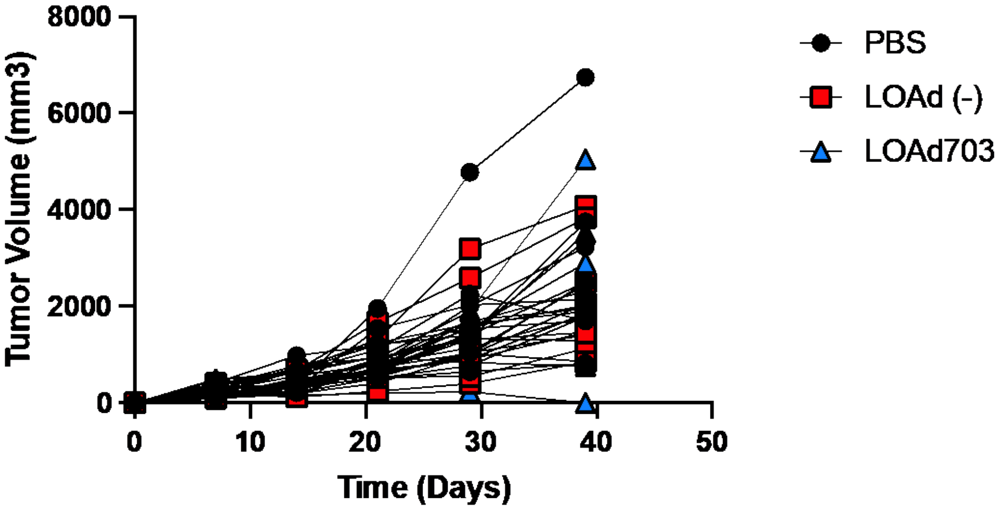

Figure S2: Treatment of Syrian hamster. Syrian hamsters are semi-permissible for adenovirus replication and were inoculated with $5x10^6$ syngeneic HP1 tumor cells. Once tumors were established, the mice were treated 4x intra-/peri-tumorally (one treatment per week) with a LOAd virus ($2x10^{10}$ VP) without transgene cassette (LOAd(-)), with a LOAd virus expressing TMZ-CD40L and 4-1BBL driven by a CMV promoter (LOAd703) or with a phosphate buffered saline (PBS) control. The figure shows the tumor growth of all tumors separately. As the cell line does not express human CD46, the virus cannot enter into the cells and there is no difference among the groups. Hence, the virus per se will not evoke immunity that eradicates tumors.

Supplementary Figure 3

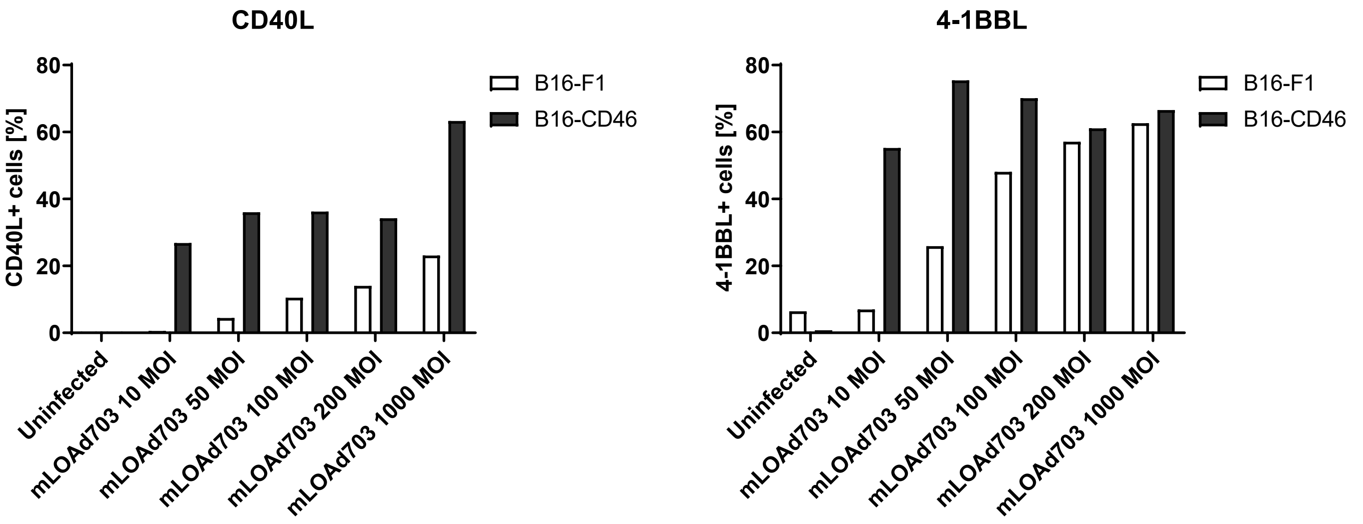

Figure S3: Transgene expression in parental B16-F1 and B16-CD46 cells. B16-F1 and B16-CD46 cells were infected with mLOAd703 *in vitro* with ascending multiplicity of infection (MOI; virus to cell ratio) ranging from 10-1000 MOI. After 48 hours, the cells were analyzed for their expression of CD40L and 4-1BBL transgenes with flow cytometry. White and black bars show the percentage of positive B16-F1 and B16-CD46 cells, respectively.

Supplementary Figure 4

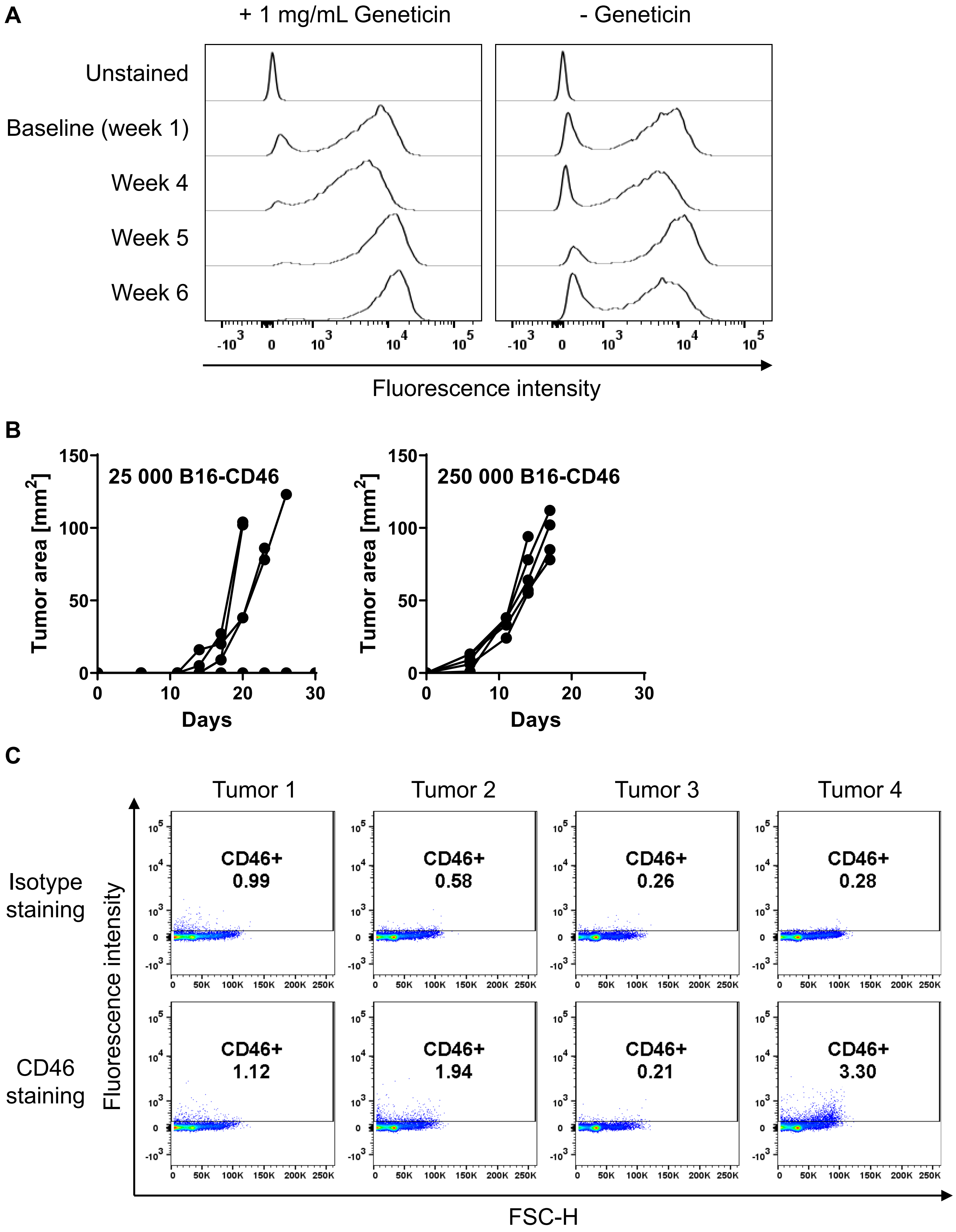

Figure S4: B16-CD46: CD46 expression and tumor growth *in vivo*. B16-CD46 cells were cultured *in vitro* with or without 1 mg/mL of the selection agent Geneticin. CD46 expression was analyzed with flow cytometry at baseline and 4-6 weeks after initiation of culture (**A**). To test *in vivo* growth different amounts of B16-CD46 cells (25 000 or 250 000 cells) were injected subcutaneously and tumor growth was measured by determining the tumor area over time (**B**). Mice from the 250 000 cells group were sacrificed at day 17 and the resected tumors were analyzed for CD46 expression with flow cytometry (**C**).

Supplementary Figure 5

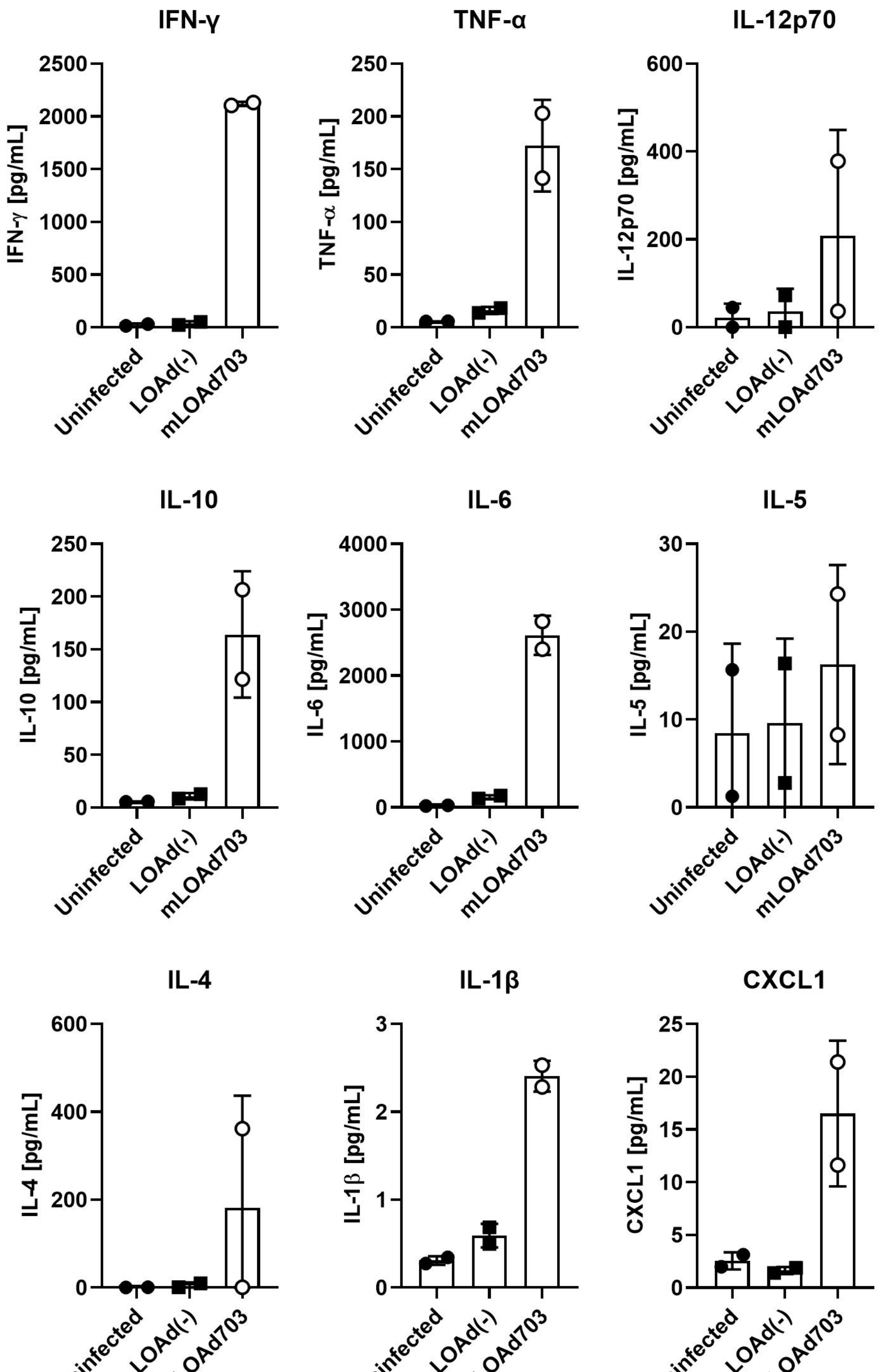

Figure S5: Activation of murine splenocyte co-cultures with mLOAd703. Splenocytes were co-cultured with murine pancreatic tumor cells (Panc02) that were either left uninfected or infected with a control virus lacking transgenes (LOAd(-)) or mLOAd703 expressing murine CD40L and 4-1BBL. Cells were co-cultured in a ratio of 10:1 (splenocytes : tumor cells) and 25 IU/mL of IL-2 was added to the cultures. After 72 hours, cell culture supernatants were removed and analyzed for the expression of cytokines with MSD V-PLEX Proinflammatory Panel 1. Bar graphs show the concentration of the respective cytokines in pg/mL. n=2

Supplementary Figure 6

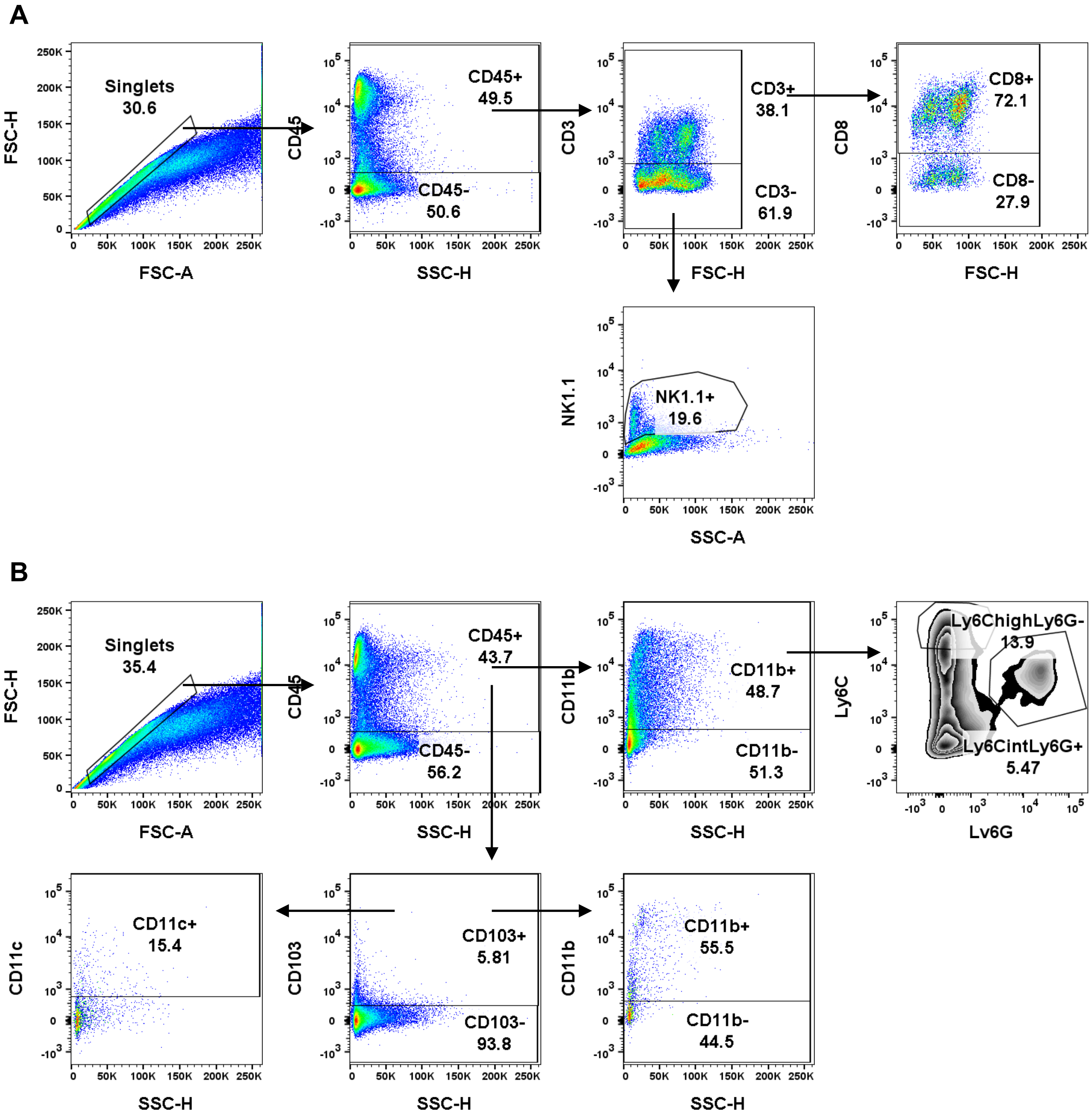

Figure S6: Flow cytometry gating strategy for tumor biopsy analysis. For analysis of T and NK cells, singlets were gated based on forward scatter (FSC)-A vs FSC-H. Single cells were gated for immune cells based on CD45 expression. CD3+ cells were gated on CD8+ and CD3- cells were analyzed for NK1.1 expression (**A**). Likewise, myeloid cells were first gated on singlets (FSC-A vs FSC-H) and immune cells were gated out based on CD45 expression. Immune cells were further gated on CD11b and CD103 expression. Myeloid derived suppressor cells were gated from the CD11b+ population based on their expression of Ly6C and Ly6G. CD103+ cells were analyzed for their expression of CD11b or CD11c (**B**).

Supplementary Figure 7

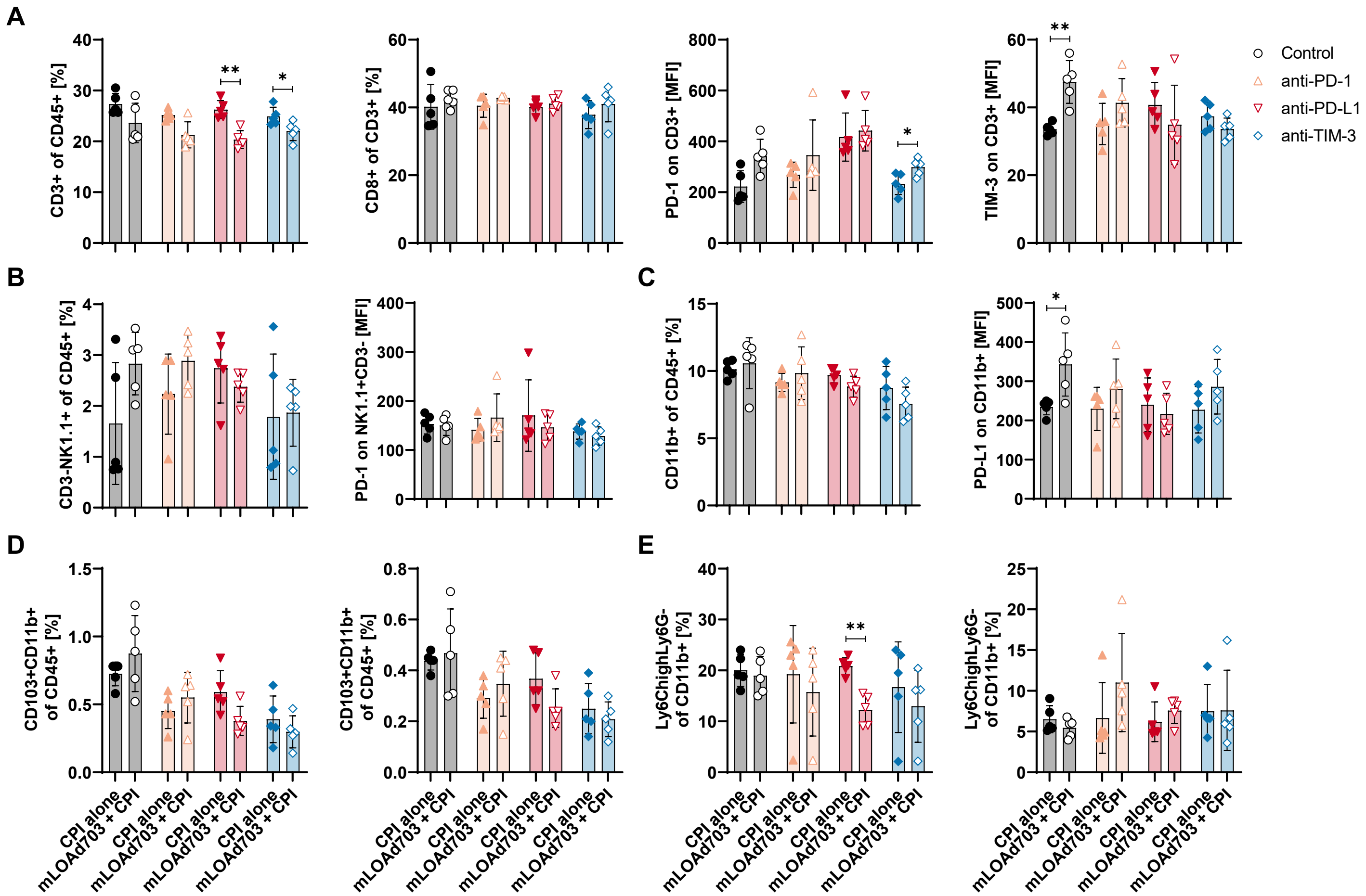

Figure S7: Immune cell infiltration in spleen biopsies. B16-CD46 cells (2x10^5) were injected subcutaneously in syngeneic C57BL/6J mice (n=5 per group). Treatments were initiated five days post tumor injection. Mice were treated either alone with mLOAd703 (i.t. 1x109 FFU/mouse), anti-PD-1, anti-PD-L1, anti-TIM-3 or IgG1/IgG2b isotype control antibodies (i.p. 100 µg/mouse) or treated with the combination of mLOAd703 with checkpoint inhibitors (CPI) for a total of three treatments. One day after the third treatment (day 13), mice were sacrificed for biopsies. Spleens were processed to single cell suspensions and analyzed with flow cytometry for immune cells: T cells (**A**), NK cells (**B**), myeloid cells (**C**), CD103+ DCs (**D**), MDSCs (**E**). Bar graphs show mean ± SD (n=5). Statistical differences between CPI alone and mLOAd703 combination treatment were calculated with Mann-Whitney test (*p<0.05, **p<0.01).

Supplementary Figure 8

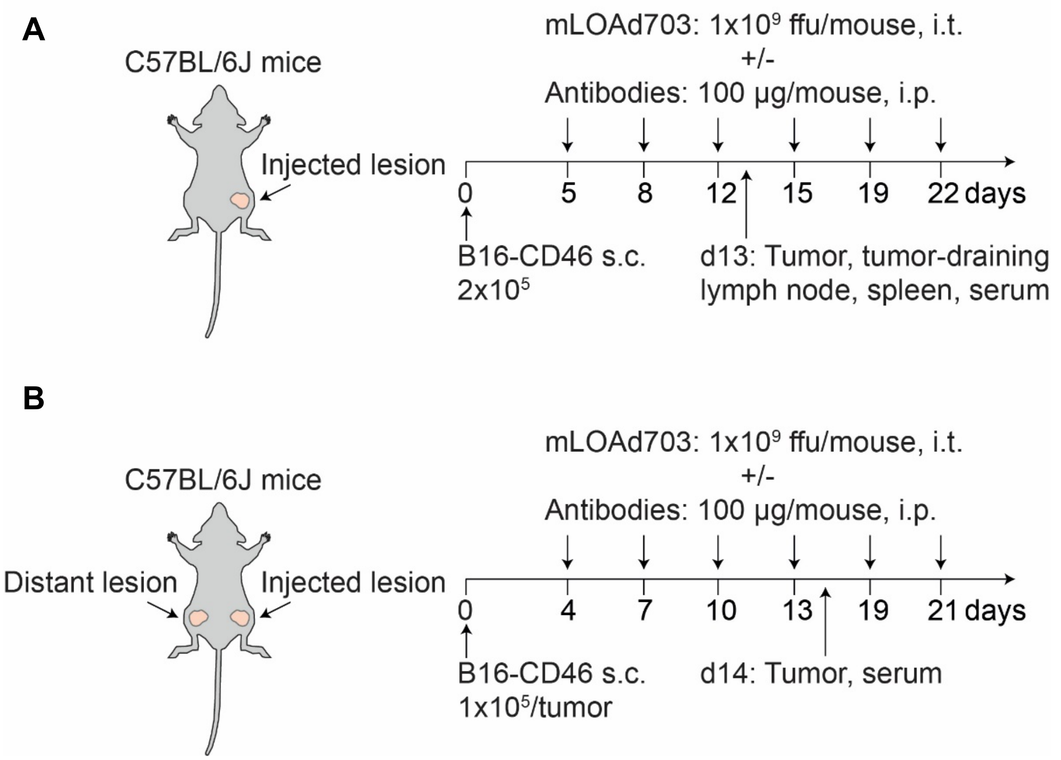

Figure S8: Experimental timeline for *in vivo* studies. For experiments with single tumor lesions, B16-CD46 cells ($2x10^5$) were injected subcutaneously in one flank of syngeneic C57BL/6J mice (n=5 per group). Treatments were initiated five days post tumor injection. Mice were treated either alone with mLOAd703 (i.t. 1x109 FFU/mouse), anti-PD-1, anti-PD-L1, anti-TIM-3 or IgG1/IgG2b isotype control antibodies (i.p. 100 µg/mouse) or treated with the combination of mLOAd703 with checkpoint inhibitors for a total of three or six treatments for biopsy or tumor growth analysis, respectively. For biopsy analysis, mice were sacrificed one day after the third treatment (day 13) (**A**). For the twin-tumor experiment, B16-CD46 cells ($1x10^5$) were injected subcutaneously at the same time in both flanks of syngeneic C57BL/6J mice (n=10 per group). Treatments were initiated four days post tumor injection. Mice were treated either with IgG2a/IgG2b isotype control antibodies, anti-PD-1, anti-PD-L1, anti-TIM-3 (i.p. 100 µg/mouse), mLOAd703 (i.t. $1x10^9$ FFU/mouse) or with the combination of mLOAd703 with checkpoint inhibition antibodies for a total of six treatments. mLOAd703 was administered intratumorally always in the same tumor lesion. One day after the fourth treatment (day 14), 5 mice per group were sacrificed for tumor biopsies and serum collection and the remaining mice were followed for tumor growth (**B**).
